# Trusted Measurement Model Based on Multitenant Behaviors

**DOI:** 10.1155/2014/384967

**Published:** 2014-03-30

**Authors:** Zhen-Hu Ning, Chang-Xiang Shen, Yong Zhao, Peng Liang

**Affiliations:** College of Computer Science, Beijing University of Technology, Beijing 100124, China

## Abstract

With a fast growing pervasive computing, especially cloud computing, the behaviour measurement is at the core and plays a vital role. A new behaviour measurement tailored for Multitenants in cloud computing is needed urgently to fundamentally establish trust relationship. Based on our previous research, we propose an improved trust relationship scheme which captures the world of cloud computing where multitenants share the same physical computing platform. Here, we first present the related work on multitenant behaviour; secondly, we give the scheme of behaviour measurement where decoupling of multitenants is taken into account; thirdly, we explicitly explain our decoupling algorithm for multitenants; fourthly, we introduce a new way of similarity calculation for deviation control, which fits the coupled multitenants under study well; lastly, we design the experiments to test our scheme.

## 1. Introduction

Cloud computing has recently attracted an important attention and dubbed as the “next best thing” in information and communication technologies (ICT) [1]. As the intrinsic feature of cloud computing, multitenancy brings sharing concept to almost all information technologies such as sharing computing resources, sharing storage resources, and sharing network. Coresident clients might have no preestablished trust relationship and might have no knowledge of the existence or identities of other clients. In such a setting, if one of the coresidents maybe attacks the other coresidents it will be much easier to succeed and be difficult to detect. Therefore, this risk incurred by trusted measurement of multitenant is a barrier to acceptance of cloud computing. Actually cloud computing system, such as Amazon's Elastic Compute Cloud (EC2), Microsoft's Azure, and Rackspace's Mosso, is a large scale system which is studied in cybernetics long before. Here we leverage the generalized predictive control affiliated to cybernetics to solve the problem of behavior measurement of multitenants on the same physical server brought by the new paradigm of cloud computing.

## 2. Background

This section consists of two parts: one is multitenancy threat; the other is the brief introduction of generalized predictive control.

### 2.1. Multitenancy Threat

It is important to consider the unique security risks introduced by multitenancy as intrinsic of the new paradigm of cloud computing in order to be able to derive adequate security solutions. As more and more applications become exported to third-party compute clouds, it becomes increasingly important to quantify any threats to confidentiality that exist in this setting [2, 3]. An obvious threat to these consumers of cloud computing is malicious behavior by the cloud provider, who is certainly in a position to violate customer confidentiality or integrity. However, this is a known risk with obvious analogs in virtually any industry practicing outsourcing. In this work, we consider the provider and its infrastructure to be trusted. This also means we do not consider attacks that rely upon subverting a cloud's administrative functions, via insider abuse or vulnerabilities in the cloud management systems (e.g., virtual machine monitors).

In our threat model, adversaries are non-provider-affiliated malicious parties. Victims are multitenants running confidentiality-requiring services in the cloud. A traditional threat in such a setting is direct compromise, where an attacker attempts remote exploitation of vulnerabilities in the software running on the system. Of course, this threat exists for cloud applications as well. These kinds of attacks (while important) are a known threat and the risks they present are understood.

We instead focus on where third-party cloud computing gives attackers novel abilities, implicitly expanding the attack surface of the victim. We assume that, like any customer, a malicious party can run and control many instances in the cloud, simply by contracting for them. Further, Based on the fact the economies offered by third-party compute clouds derive from multiplexing physical infrastructure, we assume (and later validate) that attacker's instances might even run on the same physical hardware as potential victims. From this vantage, an attacker might manipulate shared physical resources (e.g., CPU caches, branch target buffers, network queues, etc.) to learn otherwise confidential information.

### 2.2. Generalized Predictive Control

In general sense, predictive control, regardless of various algorithms, is based on the following three basic principles [4].

(*1) Predictive Model*. Predictive control is also referred to as model-based control where this model is referred to as predictive model. The predictive model can predict the future output of the object based on historical information and input. And the predictive model does not emphasize its structure but emphasizes the function of the model. Therefore, the traditional model such as equation of state and the transfer function can be used as a predictive model. Similarly, nonparametric model such as step response and impulse response can also be used directly as a predictive model. 

(*2) Rolling Optimization*. Predictive control is an optimal control algorithm, which determines the future action through an optimal performance index. However, the optimization studied in predictive control is different from optimal control in the traditional sense, and the subtle difference is that optimization in the predictive control is a rolling optimization within the limited time. At each sampling instant, the optimization performance indicators relate only to a limited time since the right moment. Until the next sampling instant, this optimization period moves forward. At different instants, the relative forms of optimization performance indicators are the same, but its absolute form, that is, containing time area, is different. Therefore, during predictive control, optimization is not offline conducted only once but repeated online, which is the core of rolling optimization, that is, the fundamental characteristics of optimal control here is different from the traditional ones. 

(*3) Feedback Correction*. Predictive control is a closed-loop control algorithm, where a series of further control actions can be ascertained by optimization. Predictive control does not perform all these actions but perform the present action. So that the deviation from the ideal state can be avoided; this is resulted from either the model mismatch or environmental interference. Until the next sampling time, the first is to detect the actual output of the object; the second is to take advantage of this real-time information to correct the prediction based on the model; and the final is to conduct the new optimization. Therefore, the optimization of the predictive control is not only based on the model, but also the feedback information, which constitutes a closed-loop optimization.

## 3. Related Work

There exist several measurement models such as Tripwire [5], AEGIS [6], and trusted box [7], the trust chain model proposed by the TCG (trusted computing group). These models focus on different measurement aspects of the system or file program, but these approaches belong to static integrity measurement of the resource. As a result, they cannot consider the dynamic trustworthiness in the system.

Further the researchers put forward the following schemes to realize dynamic measurement. In [8], there is a coprocessor-based kernel integrity monitor. The monitor periodically checks system memory and detects whether malicious programs change the host system kernel. Binding instructions and data (BIND) binds with the data and the corresponding block of process in order to provide a basis for the verification side to trace data processing. However, it cannot cope with many attacks when the system is running [9]. Policy reduced integrity measurement architecture (PRIMA) focuses on the flow of information when the system is running [10], but the model trusts flow of information which comes from the trusted subjects in mandatory access control (MAC). However, it is still a role-based privilege. The measure mode is too simple and does not conform to the definition of definition of trust. Behavior based trustworthiness attestation mode (BTAM) is trusted proof model based on the behavior of the system [11]. This model firstly determines whether the system behavior is related to trustworthiness of platform state. For a large number of behaviors that cannot be determined, this model has not yet given the solution. Therefore, the dynamic trusted measure theory and technology is an urgent need for the development of cloud computing [12].

Gong [13] firstly introduces generalized prediction control theory to analyze and measure the tenants' behaviour in the information system. The novel scheme greatly increases the trustworthiness and security of information system and opens a new direction towards behaviour measurement [13]. However, the new features mentioned above brought by the cloud computing were not considered and studied. This paper is to improve that model and to adapt the new feature of multitenancy brought by cloud computing.

## 4. Model Design

**Figure 1:**
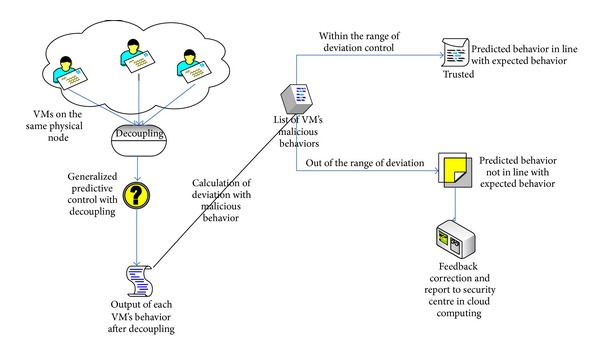
Behavior measurement model of virtual machine.

Traditional authorization and authentication are to solve the main problem whether the user's identity is trusted, while they are ineffective to solve whether the user's behavior is trusted. The original drive to promote the change of system status is the behavior [14]. Therefore, the trusted measurement of the behavior is more precise than the trusted measurement of the identity when it comes to reflect the trustworthiness of the system. The design of our model is consistent with the trustworthiness defined by Trusted Computing Group (TCG); that is, it is defined as trusted if the behavior can be expected [13]. According to this definition, we propose a measurement model for virtual machine behavior shown in Figure 1.

The first step: the characteristics of the shared resources in cloud computing brings the advantages while leading to security problems. So it is necessary to conduct decoupling control over the behavior of the virtual machines on the same physical platform. Illustratively, the decoupling control aims to simplify the control over many virtual machines sharing resources of the same physical computing node into a lot of individual control loops for each virtual machine corresponding to individual customers.

The second step: according to the decoupling control algorithm, the inputs and outputs of several virtual machines in the same physical computer can be decoupled. The decoupled inputs and outputs of appropriate virtual machine can be controlled by the generalized predictive control algorithm here. Specifically, through the past and present behavior of the virtual machine, the further behavior can be predicted.

The third step: to match predicted behavior with characteristics list of malicious behaviors so as to obtain the similarity value/deviation value. If the deviation value is less than the threshold value predetermined by the system, then the behavior is trusted, otherwise it is an untrusted behavior.

## 5. Model Implementation

The multiple tenants studied here refer to the ones who share the same physical resource such as network card and bandwidth. Due to the multitenancy sharing, the cloud computing becomes much more complicated. In order to better predict the tenant's complicated behaviors, we utilize the multiple variable generalized predictive control to capture those behaviors. In this section, firstly we depict the cloud computing system in the view of generalized predictive control; secondly, we present the description of behaviors in cloud computing; thirdly, we introduce the establishment of list of malicious tenants' behaviors; fourthly, we give decoupling algorithm for multitenant behaviors both in private and public clouds using generalized predictive control without coupling; fifthly, we give the similarity calculation used in our scheme for deviation control to confirm whether the suspected behavior is trusted or not finally.

### 5.1. Description of Controlled Object

**Figure 2:**
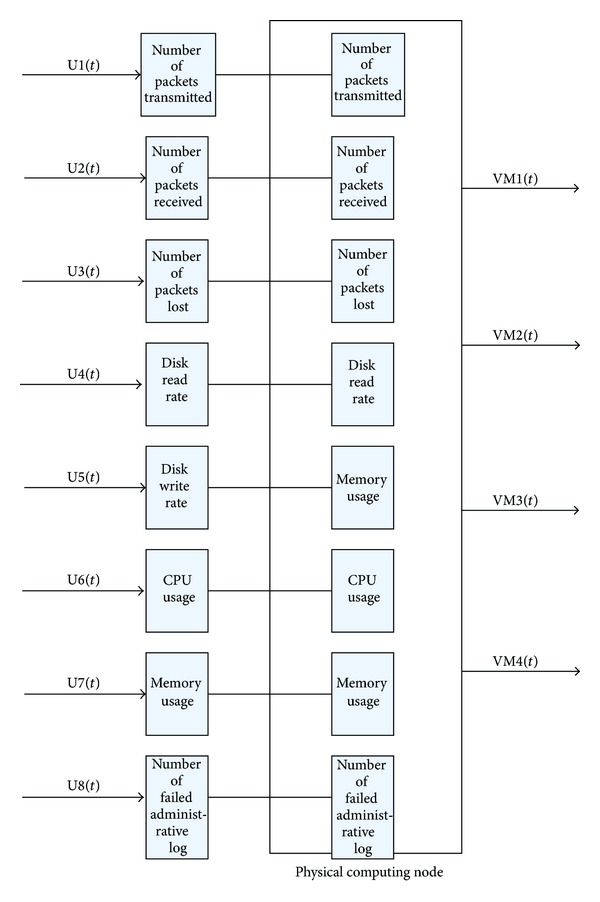
Description of physical computing nodes in the view of generalized predictive control.

From the view of control theory, the physical computing nodes where several virtual machines colocate can be taken as a multi-input, multioutput information flow control system.  Figure 2 shows a physical computing node colocated by four virtual machines from the perspective of the generalized predictive control theory. Eight behavioral measurement points are as input of the information system; the outputs are four virtual machines captured by eight behavioral measurement points, which are in line with the appropriate expectation, respectively. Each virtual machine is one of outputs of the entire physical computing node, while all four virtual machines are equal to total inputs of the entire physical compute node, such that the total traffic of all four virtual machines should be equal to the traffic of physical computing nodes.

### 5.2. Description of Tenant's Behavior

There exists monitoring components in virtualized trusted computing platform based on dual-system architecture proposed by our research team. These monitoring components can identify measurement indicators of the behavior performance of virtual machine. There are several commonly used monitoring components as follows: (1) memory and CPU monitor: to monitor memory usage and CPU call rate and report monitoring results to the behavioral data collector; (2) port monitor and message analyzer: responsible for monitoring all open TCP or UDP ports of compute nodes and capturing and analyzing communication message packet of the suspicious port. So that we can determine the role of the suspicious port and the corresponding process behavior of this port. If a suspicious user process is found to monitor a suspicious port and to communicate the message frequently, it is necessary to temporarily suspend the implementation of the process and to report to the Cloud Security Management Center; (3) network traffic detector: its role is to monitor the flow of network communication, in particular, the network traffic coming out of a virtual machine. Each virtual node has been deployed the monitor, so that both the denial of service attacks and the worm can be monitored and found. As a matter of fact, DoS and worm attacks will lead to a sharp rise in network traffic. If it is found that a virtual machine computing task unconventionally and frequently sends out a lot of the packages with the same content, this task needs to be suspended, that is, to prevent the execution of the virtual machine user tasks, and then to be reported to the Cloud Security Management Center.

In the cloud computing model, we studied the related results conducted by both foreign researchers such as Khorshed et al. [15] and local researchers such as Li et al. [16]; we choose the following to depict the virtual machine behavior, which is the number of transmitted packets, the number of received packets, the number of lost packets, disk read speed, disk write speed, memory usage, CPU usage, and the number of failed login attempts. Here, these eight performance indicators are named as measurement point, abbreviated as MP. In this paper, the behavior measurement vector of running virtual machine consists of the aforementioned 8 measurement points, see Table 1.

### 5.3. List of Tenant's Malicious Behaviors

The researchers from University of California, San Diego, and the Massachusetts Institute of Technology, Cambridge University [17] conducted a thorough experimental study on Amazon's Elastic Compute Cloud [18]. The results show that the cloud infrastructure can be mapped out, and the position of a specific virtual machine can be located. They also point out that the aforementioned information can be exploited to make side channel attacks so as to collect the information of the target virtual machine located on the same physical machine. In a recent study, Rocha and Correia [19] investigated how malicious insiders steal confidential data and demonstrated these attacks using the video and showing insiders can easily obtain passwords, encryption keys, and documents. Chonka et al. [20] reproduced the scenario of some recent attacks happening in the cloud computing and demonstrated how the HTTP-DOS and XML DoS occur in the cloud computing.

Khorshed et al. found that there exists some common factor behind these attack models [17–19], because all the attackers use a similar attack tools and follow a certain attack process. Khorshed et al. firstly collected relevant attack tools such as Hping, socket programming, httping Unix shell script, and side channel attacks. Next they collected a variety of attack scenarios related to network security by browsing relevant website and blog, such as Danchev [21] and Grossman [22] as well as their research work [23–25], and then generated attack script using the aforementioned documents.

Based on above steps, Khorshed et al. designed the experiment to collect data in the cloud computing environment. The type of data will determine the kind of data collection tools. In the attack scenario, most common data types are as follows 8 performance indicators such as the number of transmitted and received data packets, processing time, the round-trip time, and CPU usage. Khorshed et al. adopted machine learning techniques to classify the attacks related to malicious use of resources in the cloud computing. Through a large number of experiments, they obtained 8 measurement points of behavioral performance such as the number of transmitted packets, the number of received packets, the number of lost packets, disk read speed, disk write speed, memory usage, CPU usage, and the number of failed login attempts. Further, they concluded the behavioral characteristics of the classic attack in conduction of eight measurement points [26].

### 5.4. Decoupling Algorithm

To maximize efficiency, multiple VMs, one VM corresponding to one tenant, may be simultaneously assigned to be executed on the same physical server, which is supported by virtualization technology. As a result, tenants share the physical resources (e.g., CPU caches, branch target buffers, network queues, etc.) to accomplish their computation tasks. From the angle of generalized predictive control (GPC), cloud computing system under study corresponds to multiple inputs and multiple outputs system in cybernetics which is different from the single input and output system that is studied in [13]. The essential difference is the coupling between tenants on the same physical server, which should be studied thoroughly. In this section, first we use GPC theory to capture the multitenant behavior and then to derive the decoupling algorithms that is shown at the end of this part.

The multitenant's behavior in cloud computing can be described by

(1) $$\begin{array}{c} A\left(z^{-1}\right)y\left(t\right)=D\left(z^{-1}\right)B\left(z^{-1}\right)u\left(t-1\right)+\frac{C\left(z^{-1}\right)\xi \left(t\right)}{\Delta }, \end{array}$$

where *A*(*z* ^−1^) = *I* + *A*_1_ *z* ^−1^ + ⋯+*A*_*n*_*A*__ *z* ^−*n*_*A*_^,  *B*(*z* ^−1^) = *I* + *B*_1_ *z* ^−1^ + ⋯+*B*_*n*_*B*__ *z* ^−*n*_*B*_^,  *D*(*z* ^−1^) = diag⁡(*z* ^−*k*_*s*_^),  Δ = diag⁡(1 − *z* ^−1^).

{*u*(*t*)} and {*y*(*t*)} indicate coresident tenants' inputs and outputs. *ξ*(*t*) is *m*-dimension independent random disturbance vector, and its mean value and variance are zero and *σI*, respectively. Without loss of generality, suppose *A*(*z* ^−1^) is diagonal matrix.

*B*(*z* ^−1^) is divided into two parts, namely,

(2) $$\begin{array}{c} B\left(z^{-1}\right)=\bar{B}\left(z^{-1}\right)+\tilde{B}\left(z^{-1}\right), \end{array}$$

where $\bar{B}(z^{-1})$ is diagonal matrix polynomials and $\tilde{B}(z^{-1})$ is a matrix whose diagonal is zero. Equation (2) indicates that $\bar{B}(z^{-1})$ is the direct relation between tenant's inputs and outputs, and $\tilde{B}(z^{-1})$ is the mutual coupling part of communication channel.

Using (1) and (2), we have

(3) A(z−1)Δy(t)=D(z−1)B¯(z−1)Δu(t−1)+D(z−1)×B~(z−1)Δu(t−1)+C(z−1)ξ(t).

Performance index function is as follows:

(4) J=ξ·{∑j=1N||ϕ(t+j)−rjω(t+j)+Sj~(z−1)Δu(t+j−1)||Q2     +∑j=1N||Δu(t+j−i)||λj2},

where

(5) $$\begin{array}{c} \phi \left(t+j\right)=D\left(z\right)\Delta y\left(t+j\right) \end{array}$$

indicates generalized outputs, *D*(*z*) indicates the inverse of *D*(*z* ^−1^), *ω*(*t* + *j*) is fixed vector, ||*X*||_*Q*_^2^ indicates *X* ^*T*^ *QX*, and *Q* is symmetric positive definite matrix. There is no such $\tilde{S_{j}}\left(z^{-1}\right)u\left(t+j-1\right)$, part of (4), in the performance index of common generalized prediction control. $\tilde{S_{j}}(z^{-1})$ is a matrix polynomial whose diagonal is zero and $\tilde{S_{j}}(z^{-1})$ can be used to eliminate the coupling effect between channels. Similarly, weighted constant matrix *λ*_*i*_ can be divided into two $\bar{\lambda_{j}}$ and $\tilde{\lambda_{j}}$; $\bar{\lambda_{j}}$ is a diagonal matrix and $\tilde{\lambda_{j}}$ is a matrix whose diagonal is zero; the function of $\tilde{\lambda_{j}}$ is the same as that of $\tilde{S_{j}}(z^{-1})$.

We use the methods in [27] to achieve the decoupling algorithm.

Define Diophantine equation:

(6) $$\begin{array}{c} I=F_{j}\left(z^{-1}\right)A\left(z^{-1}\right)+z^{-j}D\left(z^{-1}\right)G_{j}\left(z^{-1}\right), \end{array}$$

where *F*_*j*_(*z* ^−1^) = *I* + *F*_1_^*j*^ *z* ^−1^ + ⋯+*F*_*n*_*D*_+*j*−1_^*j*^ *z* ^−*n*_*D*_−*j*+1^,  *G*_*j*_(*z* ^−1^) = *G*_0_^*j*^ + *G*_1_^*j*^ *z* ^−1^ + ⋯+*G*_*n*_*A*_−1_^*j*^ *z* ^−*n*_*A*_+1^.

Since *A*(*z* ^−1^) and *D*(*z* ^−1^) are diagonal matrix, *F*_*j*_(*z* ^−1^) and *G*_*j*_(*z* ^−1^) are diagonal matrix as well. Equation (6) is left multiplied with *D*(*z* ^−1^):

(7) $$\begin{array}{c} D\left(z\right)=D\left(z\right)F_{j}\left(z^{-1}\right)A\left(z^{-1}\right)+z^{-j}G_{j}\left(z^{-1}\right). \end{array}$$

*D*(*z*)*F*_*j*_(*z* ^−1^) left multiplies with (3), and using the above formula, we obtain the following:

(8) D(z)Δy(t+j) =Fj(z−1)B¯(z−1)Δu(t+j−1)  +Fj(z−1)B~(z−1)Δu(t+j−1)+Gj(z−1)Δy(t)  +D(z)Fj(z−1)C(z−1)ξ(t+j).

Since the term *D*(*z*)*F*_*j*_(*z* ^−1^)*C*(*z* ^−1^)*ξ*(*t* + *j*) is unrelated to other terms, optimal prediction of *ϕ*(*t* + *j*) can be represented as follows:

(9) ϕ0(t+j) =Fj(z−1)B¯(z−1)Δu(t+j−1)  +Fj(z−1)B~(z−1)Δu(t+j−1)+Gj(z−1)Δy(t).

Both $F_{j}(z^{-1})\bar{B}(z^{-1})$ and $F_{j}(z^{-1})\tilde{B}(z^{-1})$ can be divided into two parts:

(10) $$\begin{array}{c} F_{j}\left(z^{-1}\right)\bar{B}\left(z^{-1}\right)=E_{j}\left(z^{-1}\right)+z^{-j}L_{j}\left(z^{-1}\right),\\ F_{j}\left(z^{-1}\right)\tilde{B}\left(z^{-1}\right)=\tilde{E_{j}}\left(z^{-1}\right)+z^{-j}\tilde{L_{j}}\left(z^{-1}\right), \end{array}$$

where

(11) Ej(z−1)=∑i=1jEijz−i,  Lj(z−1)=∑i=1nD+nB−1Lijz−i,E~j(z−1)=∑i=1jE~ijz−i,  L~j(z−1)=∑i=1nD+nB−1L~ijz−i.

Equation (9) can be represented as

(12) ϕ0(t+j)=Ej(z−1)Δu(t+j−1)+Ej~(z−1)Δu(t+j−1)+Gj(z−1)Δy(t)+Lj(z−1)Δu(t+j−1)+Lj~(z−1)Δu(t+j−1).

Substitute the above formula into (4), and choose $\tilde{S_{j}}(z^{-1})$ that satisfies

(13) Sj~(z−1)Δu(t+j−1)+Ej~(z−1)Δu(t+j−1) +Lj~(z−1)Δu(t+j−1)=Mj~(z−1)Δu(t−1),

where ${\tilde{M}}_{j}(z^{-1})={\tilde{M}}_{0}^{j}+{\tilde{M}}_{1}^{j}z^{-1}+\cdots +{\tilde{M}}_{n_{M}}^{j}z^{-n_{M}}$ is a matrix polynomial whose diagonal is zero, so (4) can be represented as

(14) J=∑j=1N||Ej(z−1)Δu(t+j−1)+Lj(z−1)Δu(t+j−1)      +Mj~(z−1)Δu(t−1)+Gj(z−1)Δy(t)−rjω(t+j)||Q2  +∑j=1N||Δu(t+j−i)||λj2 =||EU+LΔu(t−1)+GΔy(t)+M~Δu(t−1)    −RW||I2+||U||λ2,

where *R* = diag⁡(*r*_*j*_), $\lambda =\mathrm{diag}({\bar{\lambda }}_{j})+\mathrm{diag}({\tilde{\lambda }}_{j})={\bar{\lambda }}_{j}+{\tilde{\lambda }}_{j}$,  *j* = 1,…, *N*,

(15) E=[E10E21E20⋮ENN−1ENN−2⋯EN0],U=[Δu(t),Δu(t+1),…,Δu(t+N−1)]T,W=[w(t),w(t+1),…,w(t+N−1)]T,G=[G1(z−1),G2(z−1),…,GN(z−1)]T,L=[L1(z−1),L2(z−1),…,LN(z−1)]T,M~=[M~1(z−1),M~2(z−1),…,M~N(z−1)]T.

Calculate the minimum of *J* so that we obtain

(16) U=(ETE+λ¯)−1ET×[RW−GΔy(t)−LΔu(t−1)−M~Δu(t−1)]−(ETE+λ¯)−1λ~U,

where the value of matrix $\tilde{M}$ and $\tilde{\lambda }$ can be determined by closed-loop system equation.

The first *m* rows of ${\left(E^{T}E+\bar{\lambda }\right)}^{-1}E^{T}$ are defined as *e* ^*T*^ = [*e*_1_,…, *e*_*N*_], and the first *m* rows of ${\left(E^{T}E+\bar{\lambda }\right)}^{-1}$ are defined as *h* ^*T*^ = [*h*_1_,…, *h*_*N*_].

*u*(*t*) can be represented as

(17) u(t)=[e1,…,eN]×[RW−G(t)−LΔu(t−1)−M~Δu(t−1)]−[h1λ1~+⋯+hNλN~zN−1]Δu(t).

Substituting above formula into (3), we obtain the closed-loop system equation:

(18) {[I+z−1(e1L1+e2L2+⋯+eNLNzN−1)]A  +z−1DB¯[e1G1+⋯+eNGN]}Δy(t)  =DB¯[e1r1+e2r2z+⋯+eNrNzN−1]ω(t)   −DT~Δu(t−1)+VCξ(t),

where $\tilde{T}$ indicates the mutual coupling part

(19) T~=B¯[z−1(e1M1~+⋯+eNMN~)  +(h1λ1~+⋯+hNλN~zN−1)]−[I+z−1(e1L1+⋯+eNLN)]B~.

According to (18), the coupling of closed-loop system is decoupled if and only if $\tilde{T}=0$. Because the number of variables is less than that of equations, both $\tilde{M_{j}}(z^{-1})$ and $\tilde{\lambda_{j}}$ of (19) can be obtained by least squares method; consequently $\tilde{T}$ is not equal to zero exactly, and further decoupling is approximate.

Moreover, controlled object of formula (1) is CARMA model. Since there is no steady error in outputs of closed-loop system, it is necessary to determine the matrix *r*_*j*_ of the performance index (4). To be simplified, let *r*_1_ = *r*_2_ = ⋯ = *r*_*N*_ = *r*; we can obtain *r* from formula (18):

(20) r=(e1+⋯+eN)−1×{B→(1)−1[I+e1L1(1)+⋯+eNLN(1)]A(1)  + e1G1(1)+⋯+eNGN(1)}.

After substituting $\bar{\lambda_{1}}$,  $\tilde{M_{j}}$, and *r*_*j*_ into (16), the following law of decoupling space can be derived:

(21) Δu(t)=[I,0,…,0][ETE+λ¯+λ~]−1×ET[RW−GΔy(t)−LΔu(t−1)−M~Δu(t−1)].

Generalized predictive control based decoupling algorithm is as follows.

**Step 1**

*B*(*z* ^−1^) can be divided into $\bar{B}\left(z^{-1}\right)$ and $\tilde{B}\left(z^{-1}\right)$ using (2).

**Step 2**

Least square method on $\tilde{M_{j}}\left(z^{-1}\right)$ and $\tilde{\lambda }$ can be computed out using (19).

**Step 3**

*r*_*j*_ can be calculated using (20).

**Step 4**

Δ*u*(*t*) can be derived by formula (21).

*u*(*t*) is the predicted value of individual virtual machine, after decoupling, on the virtualized platform of cloud computing.

#### 5.4.1. Decoupling Algorithm for Public Cloud

The parameters used above are known in the case the user of the virtual machine is fixed, while the aforementioned algorithm with decoupling is not applicable where the users are not fixed. For example, the users in public cloud computing are not fixed, so that the parameters related to users' behavior are unknown. In such public cloud computing, it is necessary to use parameter estimation to obtain the appropriate parameters of the corresponding controlled object and then conduct the predictive control algorithm mentioned above.

Δ left multiplies with (1); we have

(22) $$\begin{array}{c} \Delta y\left(t\right)=Q\left(z^{-1}\right)Y\left(t\right)+C\left(z^{-1}\right)\xi \left(t\right), \end{array}$$

where

(23) Q(z−1)=[A(z−1)−I,G(z−1)],Y(t)=[−Δy(t−1),−Δy(t−2),…,−Δy(t−nA),  Δu(t−1),Δu(t−2),…,Δu(t−nB)].

Formula (22) is a multivariate linear equation; we solve *Q*(*z* ^−1^) and *G*(*z* ^−1^) by the least squares method.

However, *Q*(*z* ^−1^) may change slowly with time; a typical equation is

(24) $$\begin{array}{c} G\left(z^{-1},t\right)=G\left(z^{-1},t-1\right)+\frac{M\left(t-1\right)q\left(t\right)}{\rho +M{\left(t-1\right)}^{T}M\left(t-1\right)}, \end{array}$$

where 0.95 ≤ *ρ* ≤ 1 is the forgetting factor and *q*(*t*) = *y*(*t*) − *Q*(*z* ^−1^)*Y*(*t*).

Then the method to deal with (22) may become very complex.

In this paper, to solve the newest (*z* ^−1^), we introduce the least squares method with weighs.

That is, *Q*(*z* ^−1^) satisfies

(25) $$\begin{array}{c} F=\min\sum_{i=1}^{L}\lambda_{i}q^{2}\left(i\right), \end{array}$$

where *L* is the size of the sample space and *λ*_*i*_ ≥ 0 is the weight satisfying *λ*_1_ ≤ *λ*_2_ ⋯ ≤*λ*_*L*_. Let the derivative of *F* be zero. We obtain *Q*(*z* ^−1^). To reduce the error, we can construct {u*(*t*)}_1≤*t*≤*L*_, {y*(*t*)}_1≤*t*≤*L*_ as follows.

Let *k*, *L* be integers; to obtain *G*(*z* ^−1^), we choose a series of {*u*(*t*)}_1≤*t*≤*kL*_, {*y*(*t*)}_1≤*t*≤*kL*_ and construct {u*(*t*)}_1≤*t*≤*L*_, {y*(*t*)}_1≤*t*≤*L*_ as follows:

(26) $$\begin{array}{c} \Delta u^{*}\left(t\right)=\frac{1}{k}\sum_{i=1}^{k}\Delta u\left(Lt+i\right),\\ \Delta y^{*}\left(t\right)=\frac{1}{k}\sum_{i=1}^{k}\Delta y\left(Lt+i\right). \end{array}$$

Since the mean value of *ξ*(*t*) is 0, then we have

(27) $$\begin{array}{c} \frac{1}{k}\sum_{i=1}^{k}\xi \left(mt+i\right)\approx 0. \end{array}$$

Then using the least squares method with weighs on {u*(*t*)}_1≤*t*≤*L*_,{y*(*t*)}_1≤*t*≤*L*_, we obtain *Q*(*z* ^−1^).

### 5.5. Deviation Control

The behavior of the virtual machine can be mapped to a point in the space that consists of eight behavioral measurement points. The model of behavioral trusted measurement can determine whether the behavior of the virtual machine is out of security border, that is, whether the behavior is a malicious one. Mathematically, the aforementioned is to obtain the distance between two points in 8-dimensional space that consists of 8 behavior measurement points. This is actually a problem to calculate the similarity between two different objects.

Similarity calculation is widely used in the intrusion detection technology and other technologies. The typical solutions are like inner product, Dice coefficient, cosine function, and Jaccard coefficient method [28].

In this paper, gray correlation analysis is adopted to calculate the deviation value. Because the predictive value of the virtual machine behavior is unknown, the historical and present behavior of the virtual machine is consistent with the information, so that this known information and corresponding location information constitute a gray system [29]. At present, the gray system theory has been extended to many fields such as the industrial, agricultural, social, economic, energy, geology, and petroleum, successfully solving a large number of practical problems in production, living, and scientific research and making remarkable achievements. The gray relational analysis is a branch of the gray system theory.

The basic idea of gray relational analysis is to determine whether they are similar to each other by the degree of similarity of curve geometry composed of the appropriate data sequence. In terms of mathematics, gray correlation degree is used here to reflect the degree of similarity. The closer the two curves are, the greater the degree of correlation of the two corresponding data sequences is, and vice versa. When it comes to specific analysis, it is desirable to replace unlimited convergence curve with approximate convergence (data array), so as to provide a great convenience in the case of dealing with a large number of practical problems.

Combined with the characteristics of a distributed computing environment based on virtual architectures, Grey Relational Analysis is adopted in this paper, and the specific calculation steps are as follows.

(1) According to the measurement point of the behavior of the virtual machine, to create the reference sequence of a virtual machine behavior, suppose *n* data sequences can form the following matrix:

(28) (X1′,X2′,…,Xn′)=(x1′(1)x2′(1)⋯xn′(1)x1′(2)x2′(2)⋯xn′(2)⋮⋮⋮⋮x1′(8)x2′(8)⋯xn′(8)).

8 indicates the number of behavioral measurement points while *n* represents the time series:

(29) $$\begin{array}{c} X_{i}^{\prime }={\left(x_{i}^{\prime }\left(1\right),x_{i}^{\prime }\left(2\right),\ldots ,x_{i}^{\prime }\left(8\right)\right)}^{T},\;i=1,2,\ldots ,n. \end{array}$$

The data sequence is known as the reference sequence that can reflect the characteristics of the behavior of the system. The data sequence is known as comparison sequence that is composed of the factors that affect behavior of the system.

(2) The goal of the behavior of the virtual machine decides the value of the behavioral measurement point and further determines comparison sequence that has impact on the behavior of the system.

Reference data sequence should be a standard for the comparison. Here, reference data sequence comes from the list of the behavioral characteristics, seen in Table 1, written as

(30) $$\begin{array}{c} X_{0}^{\prime }=\left(x_{0}^{\prime }\left(1\right),x_{0}^{\prime }\left(2\right),\ldots ,x_{0}^{\prime }\left(8\right)\right). \end{array}$$

(3) Nondimensionalization of the reference sequence and the comparison sequence.

Due to the fact that the factors in the system have various physical meanings, the dimensions involved in the factors are different as well. As a result, it is difficult to compare the factors so as not to obtain a correct conclusion. When it comes to Grey Relational Analysis, generally it is required to carry out nondimensionalization of the appropriate data. The methodologies commonly used for nondimensionalization are as follows, for example, equalization method and the initialization method, seen in (31):

(31) xi(k)=xi′(k)(1/8)∑k=18xi′(k),  xi(k)=xi′(k)xi′(1)i=0,1,…,n;  k=1,2,…,8.

After nondimensionalization, data sequence is as follows:

(32) (X0,X1,…,Xn)=(x0(1)x1(1)⋯xn(1)x0(2)x1(2)⋯xn(2)⋮⋮⋮⋮x0(8)x1(8)⋯xn(8)).

Here the initialization method is adopted to conduct nondimensionalization.

(4) In our scheme, the comparison sequence refers to the behavioral measurement vector of the virtual machine to be measured. For every behavior of the virtual machine, the corresponding absolute difference between the comparison sequence and reference sequence is calculated,respectively; that is, |*x*_0_(*k*) − *x*_*i*_(*k*)|, where *k* = 1,…, 8; *i* = 1,…, *n*,  *n* is defined as the number of sampling values of the object to be measured during a given period.

(5) Calculate both min⁡_*i*=1_^*n*^ min⁡_*k*=1_^8^|*x*_0_(*k*) − *x*_*i*_(*k*)| and max⁡_*i*=1_^*n*^ max⁡_*k*=1_^8^|*x*_0_(*k*) − *x*_*i*_(*k*)|.

(6) Calculation of the relational coefficient through formula (33), the coefficient of the appropriate elements between every comparison sequence and reference sequence is calculated, respectively. Relational coefficient actually represents the degree of the difference between two curves in terms of geometry. Therefore, the degree of difference can reflect the degree of relationship:

(33) $$\begin{array}{c} \zeta_{i}\left(k\right)=\frac{T_{1}}{T_{2}}, \end{array}$$

where

(34) T1=min⁡i min⁡k|x0(k)−xi(k)|+ρ·max⁡i max⁡k|x0(k)−xi(k)|,T2=|x0(k)−xi(k)|+ρ·max⁡i max⁡k|x0(k)−xi(k)|,

where *ρ* is identification coefficient, 0 < *ρ* < 1, and usually *ρ* = 0.5.

(7) Calculation of the degree of relationship.

Because the relation coefficient reflects the degree of relationship between comparison sequence and reference sequence at each moment. So, obviously there is more than one value and these values are dispersed. Therefore, it is necessary to use one value to reflect all of relation coefficient values moment. Here the average value is chosen to represent the degree of relationship between the comparison sequence and the reference sequence. The corresponding formula is as follows:

(35) $$\begin{array}{c} r_{0i}=\frac{1}{m}\sum_{k=1}^{m}\zeta_{i}\left(k\right). \end{array}$$

## 6. Simulation and Results

In this paper, NetLogo simulation is the use of cloud computing mode virtual machines on the virtual platform to analyze our behavior-based trust measurement program. NetLogo is based modeling and integration of multiagent simulation environment, especially for the time evolution of complex systems modeling and simulation. The test environment is Intel Core Duo 2.36 g, and 4 G memory used NetLogo win7 runs to simulate the behavior of the virtual machine on a shared virtual platform, and we measure the effectiveness of the model checking virtual machine malicious behavior tested. Here the basic parameters are shown in Table 2.

A major function of the proposed scheme is to detect a variety of malicious behaviors of the virtual machine. To guarantee the trustworthiness of the group as much as possible, this paper uses the successful detection rate (abbreviated as MSR) of malicious behavior to reflect the detection ability of our scheme against malicious behaviors.

Within Δ*t*, suppose there are *b*(*t*) computing nodes with malicious behavior and *a*(*t*) computing nodes with trusted behavior in the system, so that *β*% can be described as follows:

(36) $$\begin{array}{c} \beta \%=\frac{b\left(t\right)}{a\left(t\right)+b\left(t\right)}. \end{array}$$

This paper will simulate the attack process of “worm” virus, and then to test the effectiveness of our scheme by detecting the behavior of the “worm” virus. As a matter of fact, worm virus has the following characteristics such as breaking into antivirus software, compromising security model of the system, and implantation of Trojan into downloader. The virus typical invasion action [30] is denoted by

(37) $$\begin{array}{c} \text{Attack}\_\text{Behavior}. \end{array}$$

According to the description of the behavior in Table 1, worm virus attacks can be abstracted as a behavioral vector:

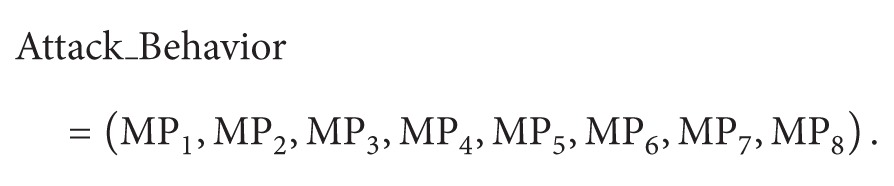
(38)


In order to verify the effectiveness of the trusted measurement method of the behavior of the virtual machine here, we take the scheme without the decoupling proposed in literature [13] as contrast. In our experiments, the initial ratios of infected virtual machines are set as 30%, 50%, and 70%, respectively. For behavioral trusted measurement both with decoupling and without decoupling based on generalized predictive control, the experimental simulation are carried out three times.

**Figure 3:**
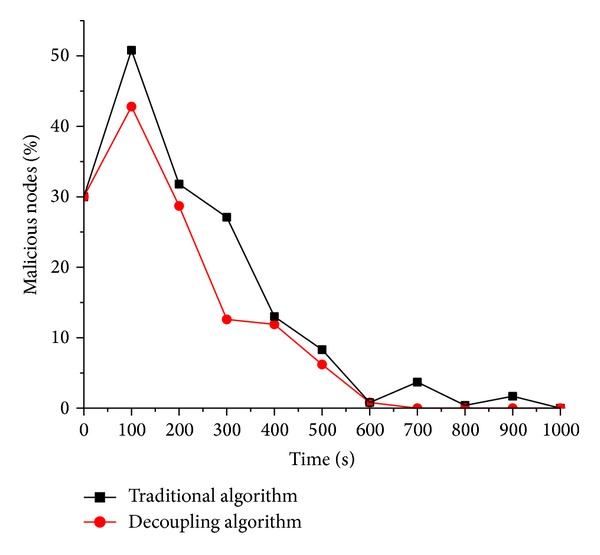
Percentage of the malicious suspicious virtual machine node is 30%.

**Figure 4:**
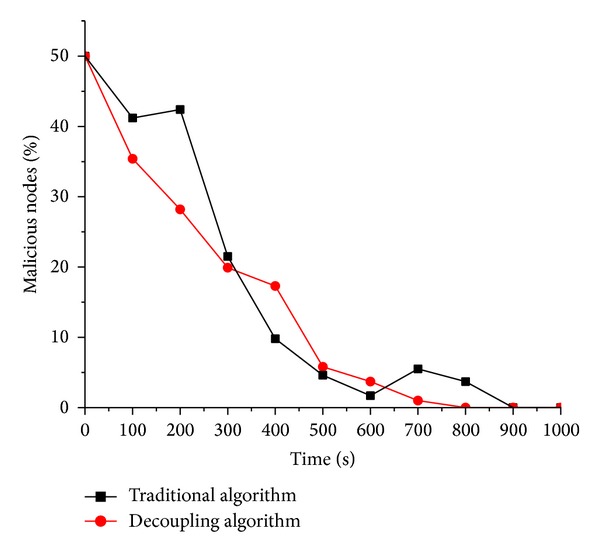
Percentage of the malicious suspicious virtual machine node is 50%.

**Figure 5:**
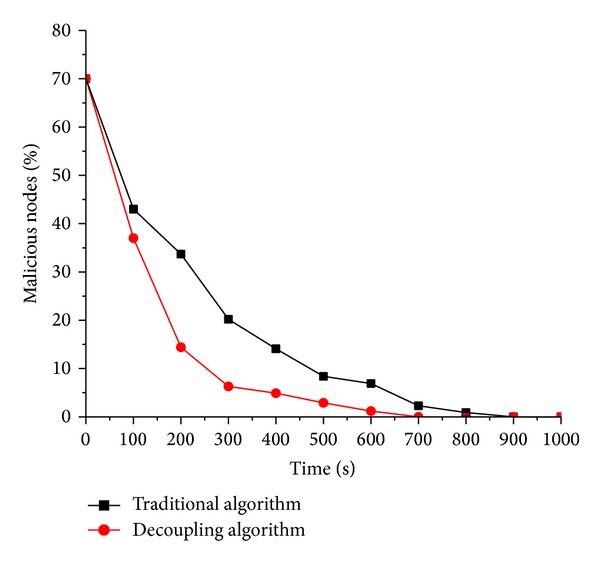
Percentage of the malicious suspicious virtual machine node is 70%.

When the percentages of malicious nodes are 30%, 50%, and 70%, the corresponding experimental results are shown from Figure 3 to Figure 5. After the analysis of Figures 3, 4, and 5, the following conclusions are summarized.

(1) Generally by the analysis of three figures, the simulation system for behavioral measurement model with decoupling can reach a steady state faster than the one without decoupling. The so-called steady state means such state that the number of the malicious nodes within the simulation system is 0. In our experiments, one of the parameters is the recovery chance that indicates the probability that infected node recovers as normal. In practical applications, finally the infected compute nodes recover as normal by various measurements, for example antivirus software. Faster to reach steady state means the corresponding scheme of behavioral trusted measurement is more accurate than the counterpart; that is, the user can detect and stop the spread of malicious worm virus timelier.

(2) In Figures 3, 4, and 5, the red line (decoupling algorithm) is almost below the black line (traditional algorithm), which indicates that, at any time, the scheme with decoupling proposed here can help accurately reflect the trusted state of the virtual machine and further take timely measurement so as to restrict the spread of the worm virus.

(3) In Figure 5, the distance between the red line (decoupling algorithm) and the black line (traditional algorithm) is larger than the previous two figures, which indicates, as the proportion of the malicious nodes in the system goes more, that the behavioral trusted measurement proposed here is better than the scheme in [13].

In summary, the experimental simulation shows that trusted measurement scheme here can effectively predict and control the behaviors of the virtual machine. So that such attack behavior that results from the abuse of the resources in cloud computing can be found timely and well restricted; that is, the security of the entire group can be well guaranteed.

## 7. Conclusion

The scheme for trusted measurement over dynamic multitenant behavior in cloud computing environment put forward here addresses the problem of resource-sharing existing in the cloud computing. By extending our previous model to the multiple tenants who share the same resource, we can further use the generalized predictive control to depict complicated behavior in cloud computing. Thanks to the advantages of generalized predictive control such as rolls optimized method and the feedback adjustment, the complicated behaviors of multitenants are well controlled. Further, the problems incurred by coupling between multitenants are solved effectively by the decoupling algorithm of generalized predictive control. As a result, the malicious behaviors between multitenants are restricted in cloud computing platform. In other words, our scheme avoids the threats introduced by multitenancy under cloud computing. In the future, we will refine our scheme and take into account the nonlinear behaviors between multiple tenants in order to deal with the behavior of tenants much more precisely.

## Figures and Tables

**Table 1:** Virtual machine behavior metric vector.

Measurement point	Measured object
MP_1_	Number of packets transmitted
MP_2_	Number of packets received
MP_3_	Number of packets lost
MP_4_	Disk read rate
MP_5_	Disk write rate
MP_6_	Memory usage
MP_7_	CPU usage
MP_8_	Number of failed administrative log on attempt

**Table 2:** Simulation parameters.

Items	Meanings
*N*	350 VMs on the physical computing node
*M*	Initial number of infected VM when virus outbreaks
Average-node-degree	Number of interacted VMs with given VM
*r*_0*i*_	Deviation between predicted behaviour and expected behaviour
Time	Running time of simulation of the system
*β*%	Percentage of malicious VMs versus the total VMs
Recovery chance	Recovery probability of the virtual computing nodes
